# Narcolepsy: a machine learning bibliometric analysis (1996–2024)

**DOI:** 10.3389/fneur.2025.1505574

**Published:** 2025-06-25

**Authors:** Minheng Zhang, Xiaodong Hu, Haiyan Wu, Haixia Fan

**Affiliations:** ^1^The First People's Hospital of Jinzhong, Jinzhong, Shanxi, China; ^2^First Hospital of Shanxi Medical University, Taiyuan, Shanxi, China

**Keywords:** narcolepsy, comorbidities, molecular mechanisms, treatment, bibliometric analysis

## Abstract

**Background:**

Narcolepsy is a rare neurological cause of chronic sleepiness. This study aimed to better understand global narcolepsy through bibliometric analysis.

**Methods:**

Articles and reviews on narcolepsy were sourced from the Web of Science Core Collection. A bibliometric analysis was performed using Microsoft Excel, Python, CiteSpace, VOSviewer, R (bibliometrix), and the Online Analysis Platform of Literature Metrology to assess publication outputs, countries, institutions, authors, journals, co-cited references, and keywords.

**Results:**

The analysis included 5,215 publications, with citations significantly increasing from 1996 to 2024. The USA led in publications, while the top institutions were Stanford University, INSERM, and Université de Montpellier. Key authors like Professors Plazzi G, Mignot E, and Dauvilliers Y greatly contributed to the field through numerous publications and high citation rates. Sleep published the most articles, followed by Sleep Medicine. Keyword analysis indicated a shift toward molecular mechanisms, comorbidities, and diagnosis. Recent interest has surged in medications for excessive daytime sleepiness, such as “Pitolisant”, “Modafinil” and “Sodium Oxybate” along with the relationship between narcolepsy and COVID-19.

**Conclusion:**

“Pitolisant,” “Modafinil,” and “Sodium Oxybate” have gained prominence in narcolepsy treatment. This study also highlights common comorbidities linked to narcolepsy, including “obstructive sleep apnea”, “epilepsy” and “atrial fibrillation” driving researchers to explore these conditions to improve the quality of life for affected individuals. However, the interactions between key neurotransmitters in narcolepsy are still unclear, and challenges remain regarding factors that complicate drug therapy efficacy, necessitating further investigation.

## 1 Introduction

Narcolepsy was initially identified in the late nineteenth century and was considered a rare and nonspecific disorder (1). By the 1960s, it had been formally recognized as a distinct neurological disease, defined by a specific set of clinical features: excessive daytime sleepiness (EDS), cataplexy, and disrupted nocturnal sleep. Narcolepsy affects individuals of all ages, including both adults and children, and has a significant impact on daily functioning, overall wellbeing, and quality of life (2). Currently, narcolepsy is classified into two forms, both characterized by EDS and positive results on multiple sleep latency tests. Type 1 narcolepsy (NT1) is distinguished by the presence of cataplexy and reduced levels of orexin-A, while Type 2 narcolepsy (NT2) lacks cataplexy and exhibits normal orexin-A levels, suggesting that despite having similar clinical phenotypes, the underlying etiologies differ significantly (3). The average time from the onset of symptoms to diagnosis is 5–15 years, and narcolepsy may remain undiagnosed in as many as half of all affected people with narcolepsy (4). Over the past two decades, substantial advancements in understanding narcolepsy have been made, primarily driven by innovative research methodologies and technological developments. These advancements have resulted in an exponential increase in the volume of scientific literature, which has facilitated the use of systematic reviews and meta-analyses to evaluate research findings both qualitatively and quantitatively (3, 5, 6). However, despite these advancements, there remains a notable scarcity of recent, comprehensive analyses aimed at providing insights into emerging research trends and key focal points within the field of narcolepsy.

Bibliometric analysis, which utilizes citation data to quantitatively assess the distribution and impact of scientific literature across various entities within a given field, has become an increasingly valuable tool for evaluating research trends and scholarly output (7–9). Such an analysis is essential to provide a clearer, more current understanding of narcolepsy-related scientific output, thereby informing evidence-based decision-making and guiding strategic research and policy development (10). To address this gap, the primary aim of this study was to assess the evolution of narcolepsy-related knowledge domains over recent decades, employing co-citation network analysis to identify seminal studies and emerging research trends. A secondary objective was to systematically map the landscape of narcolepsy research by quantifying contributions from key entities, including countries, institutions, authors, and journals. Additionally, this analysis aims to uncover existing research gaps, limitations, and unexplored opportunities, thereby providing essential insights for the formulation of future research priorities and the development of targeted policies in the field of narcolepsy.

## 2 Methods

### 2.1 Database and search strategy

This study used the Web of Science Core Collection Database (WoSCC), a widely recognized bibliometric dataset (11). The search terms included:((((((((((((((((((TS=(Narcolepsy)) OR TS=(Gelineau Syndrome)) OR TS=(Syndrome, Gelineau)) OR TS=(Gelineau's Syndrome)) OR TS=(Gelineaus Syndrome)) OR TS=(Gelineau's Syndromes)) OR TS=(Syndrome, Gelineau's)) OR TS=(Syndromes, Gelineau's)) OR TS=(Narcoleptic Syndrome)) OR TS=(Narcoleptic Syndromes)) OR TS=(Syndrome, Narcoleptic)) OR TS=(Syndromes, Narcoleptic)) OR TS=(Paroxysmal Sleep)) OR TS=(Sleep, Paroxysmal)) OR TS=(Narcolepsy-Cataplexy Syndrome)) OR TS=(Narcolepsy Cataplexy Syndrome)) OR TS=(Narcolepsy-Cataplexy Syndromes)) OR TS=(Syndrome, Narcolepsy-Cataplexy)) OR TS=(Syndromes, Narcolepsy-Cataplexy). All data were collected on August 26, 2024. We excluded non-research items such as proceedings, corrections, news items, book chapters, retracted publications, and editorials. Only English articles and reviews were retained, resulting in 5,215 studies for analysis.

### 2.2 Data processing

#### 2.2.1 CiteSpace

CiteSpace (6.2.4R, 64-bit Advanced Edition) (12) was used to analyze the data. The period covered was from January 1996 to August 2024, with a time slice of 1 year. Node types included author, institution, and keywords. For author and institution nodes, the threshold was set to the top 25 per slice without pruning. For keywords, a threshold of 25 was set with pruning using pathfinder and merged network techniques. Visual analyses generated knowledge maps of researchers, institutions, and keywords. All records retrieved from WoSCC were saved as “full records and cited references” in plain text.

#### 2.2.2 VOSviewer

Data were processed using VOSviewer 1.6.20, developed by CWTS at Leiden University (13). Full counting was used, with thresholds set based on analytical items to generate visual representations for collaborative network analysis.

#### 2.2.3 Bibliometrix

The bibliometrix R package (https://www.bibliometrix.org), developed by Dr. Massimo Aria and Corrado Cuccurullo (14), was used for historiographic analysis, tracking journal and author trends, and calculating metrics such as g-index (15), h-index (16), number of citations (NC), and publications (NP) (14).

#### 2.2.4 The other tools

Microsoft Excel 2021 (Version 16.48) was utilized for the initial data organization. Python (Version.12.6) was used to analyze and plot yearly publication trends. The Online Analysis Platform of Literature Metrology (https://bibliometric.com/) provided bibliometric analysis of citation data in an intuitive manner.

## 3 Results

### 3.1 Annual publications trends

**Figure 1:**
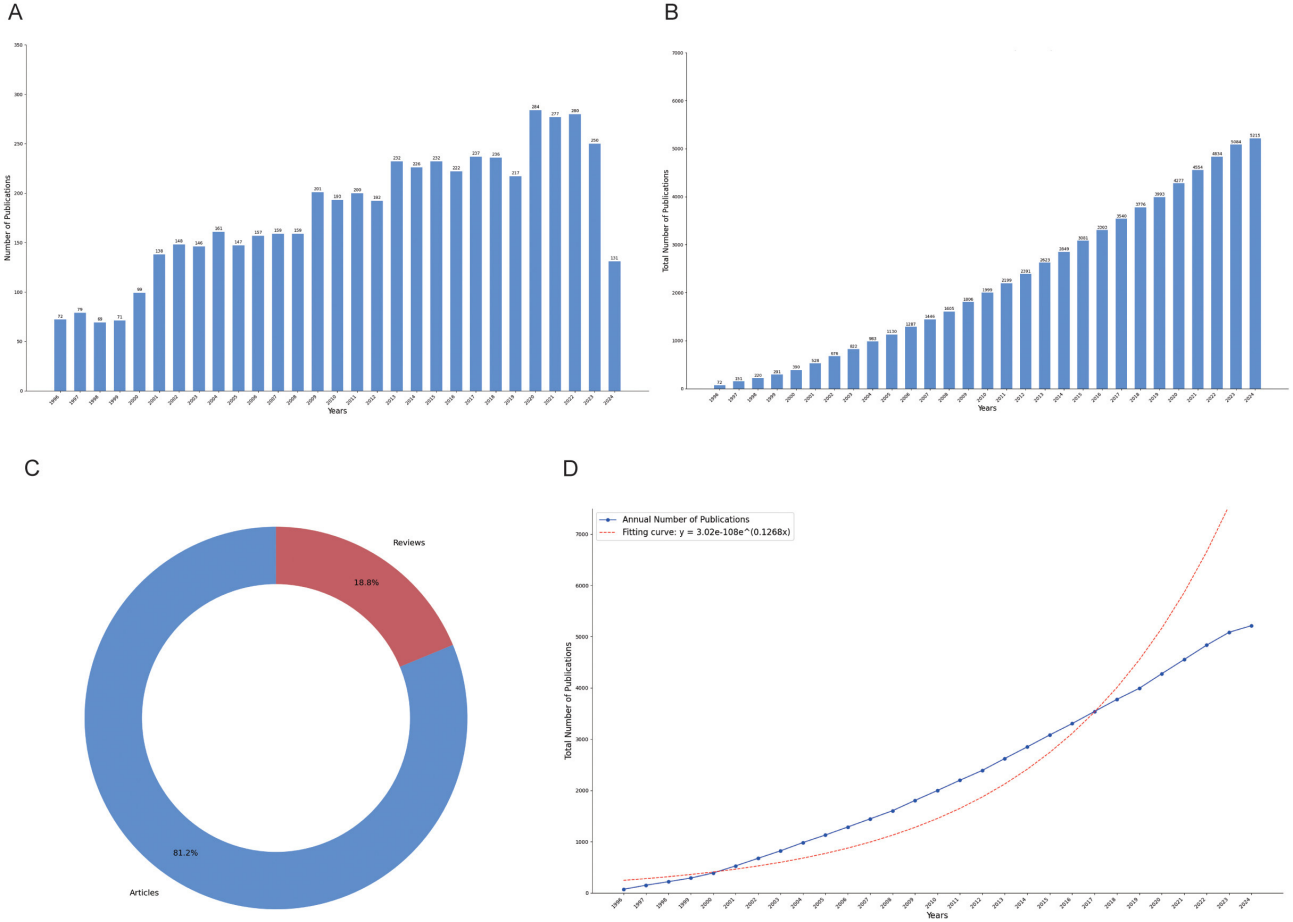
Publication trends in narcolepsy research from 1996 to 2024. **(A)** Annual publication counts; **(B)** Total publication volume; **(C)** Literature type distribution; **(D)** Price's law of cumulative growth curve fitting analysis.

Our analysis spanned from 1996 to 2024, encompassing a total of 5,215 documents. These documents represent a rich overview of the research landscape over nearly three decades, reflecting the growing volume and diversity of scholarly contributions in the field. The annual growth rate of publications was 2.96%, indicating steady expansion in research activities over the years (Figure 1A). Figure 1B depicts the cumulative total number of publications, showcasing consistent growth over time, while Figure 1C illustrates the distribution of document types, with articles (4,237) comprising 81.2% of the total and reviews (978) accounting for 18.8%. Price's Law growth curve (Figure 1D) was used to evaluate the projected vs. actual publication trends, showing a close alignment with an exponential growth pattern. A logistic regression model *y* = 3.02e−108e^∧^ (0.1268x), indicating moderate fit. Besides, a total of 17,298 unique authors contributed to the dataset, with an average of 6.05 co-authors per document. Notably, 25.7% of the publications involved international collaboration, while 366 were single-authored, reflecting both individual efforts and the dominance of collaborative research. In terms of content, 7,634 author keywords and 7,445 Keywords Plus were identified, providing insight into the thematic focus areas and emerging trends. The publications collectively contained 112,470 references, with an average citation count of 44.22 per document, pointing to the substantial academic impact of the included studies. This comprehensive dataset allows for an in-depth analysis of publication trends, collaboration networks, and thematic shifts across the years (Supplementary Table 1).

### 3.2 Distributions of countries/regions

**Figure 2:**
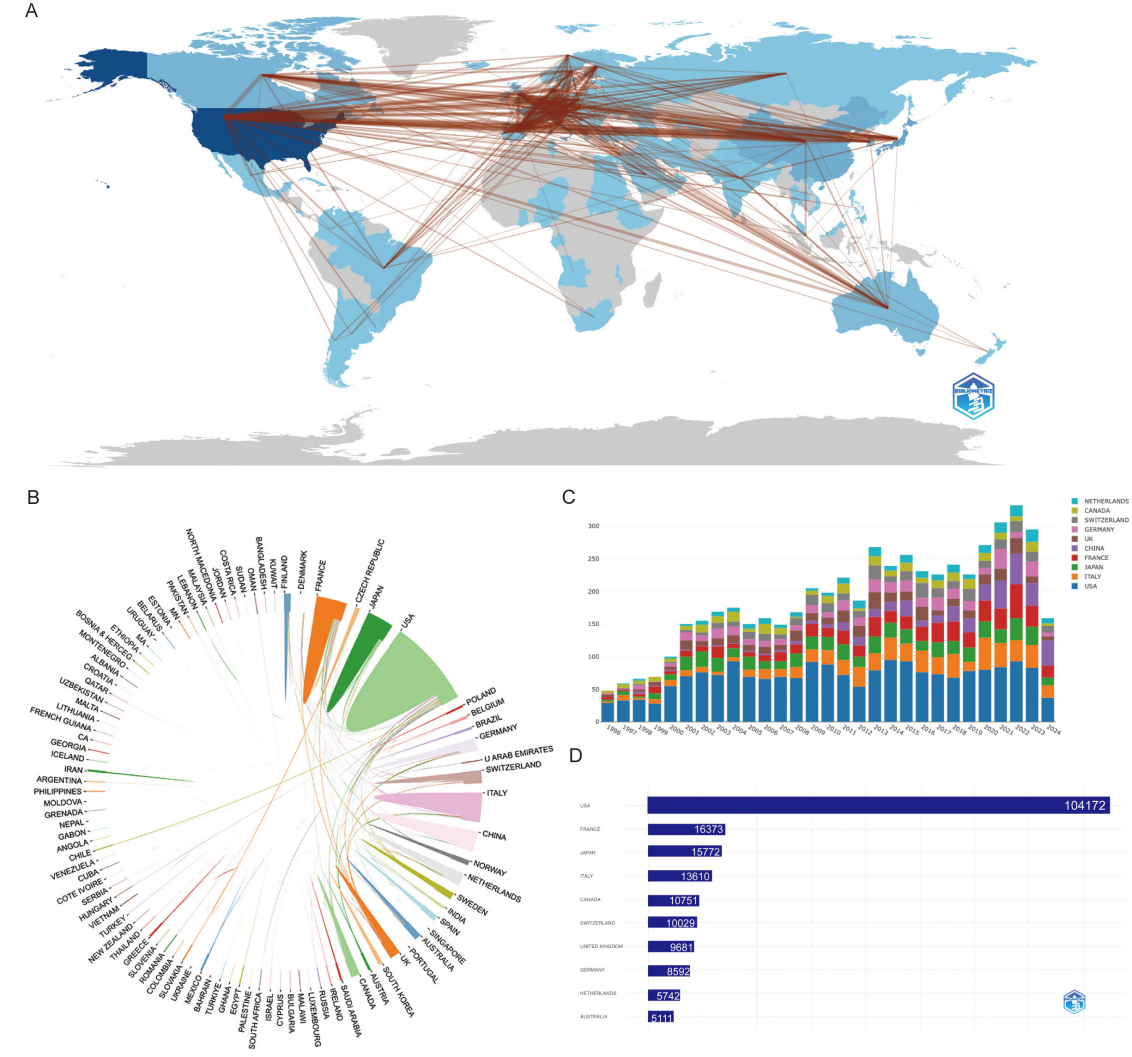
Visualization and analysis of country/region involvement in narcolepsy research. **(A)** Country/Region Collaboration Map. Darker shades of blue indicate higher collaboration rates, and the width of the link lines represents the strength of collaboration between two countries. **(B)** Network map of national research output and cooperative relations. **(C)** Distribution of countries/regions over Time. Each bar represents a country and its height corresponds to the total number of articles published. **(D)** Top 10 countries/regions by citation impact.

Figure 2A offers a comprehensive depiction of these networks, showcasing concentrated collaborations primarily among the United States, Europe, and select Asian countries. A total of 83 countries and regions are actively engaged in narcolepsy research, with the United States leading decisively, contributing 1,626 publications (31.2%). The dense web of collaboration between the United States and other nations, particularly in Europe, underscores its pivotal role in international research partnerships. European countries, including Italy (421, 8.1%), France (349, 6.7%), Germany (202, 3.9%), the United Kingdom (222, 4.3%), and Switzerland (194, 3.7%), also demonstrate substantial involvement, reflecting the region's significant contributions to the field. In Asia, China and Japan have emerged as prominent contributors, with 399 and 289 publications, respectively, indicating their expanding influence in narcolepsy research (Supplementary Table 2). The concept of betweenness centrality, which signifies the importance of a country's position within the research network, is illustrated in Figure 2B, similar to the network visualization in Figure 2A. Figure 2C shows the distribution of publications across different countries over time, with each bar representing the cumulative number of articles published by a specific country. Figure 2D presents the top 10 countries and regions by citation count, with the United States leading by a significant margin, accumulating 104,172 citations, which corresponds to an average of 64.1 citations per article. This underscores the substantial influence of U.S.-based research on the field. France, Japan, and Italy follow, with total citations of 16,373, 15,772, and 13,610, respectively, each maintaining an average citation rate between 32.3 and 46.9. Supplementary Table 2 focuses on the corresponding author contributions by country, distinguishing between single-country publications (SCP) and multiple-country publications (MCP). The United States stands out with the highest volume of research output in both SCP and MCP categories, highlighting its leadership in global scientific productivity. Notably, several countries, such as Germany, Australia, and Switzerland, exhibit a high proportion of MCPs, indicating a strong emphasis on international collaboration. This pattern suggests differing research dynamics, with nations like Germany prioritizing cross-border partnerships, while others adopt a more independent approach to research.

### 3.3 Distribution by institutions

**Figure 3:**
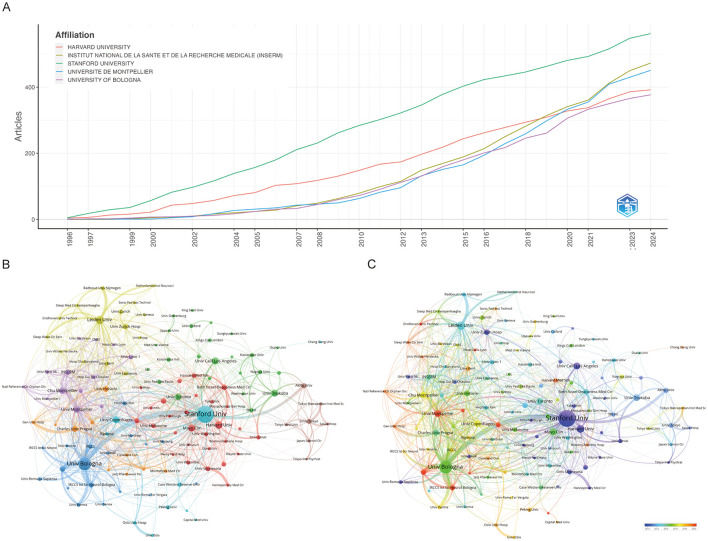
Visualization and analysis of institutional involvement in narcolepsy research. **(A)** Annual publication counts of the top 5 journals. **(B)** Institutional clustering analysis and **(C)** Timeline visualization of institutional collaborations. Node size represents the publication volume of each institution, line thickness indicates the strength of collaborative ties, and node colors distinguish between different collaborative clusters.

As shown in Figure 3A, the top five institutions by research productivity are Stanford University (562 articles), INSERM (473), Université de Montpellier (451), Harvard University (392), and the University of Bologna (377) (Supplementary Table 3). Notably, while Harvard University long held the second position, INSERM has seen a marked rise in output, surpassing Harvard in 2018 (Figure 3A). Figure 3B presents a clustering analysis of institutional collaborations using VOSviewer, revealing a complex network of interactions. Notably, Stanford University, the University of Bologna, and Leiden University emerge as central hubs, actively fostering research partnerships. Stanford, in particular, demonstrates extensive regional collaborations. Temporal cluster analysis (Figure 3C) indicates that institutions shown in purple and light blue represent long-established contributors, while those in yellow and red, such as the University of Bologna and Université de Montpellier, reflect newer or recently active institutions.

### 3.4 Distributions of authors and co-cited authors

**Figure 4:**
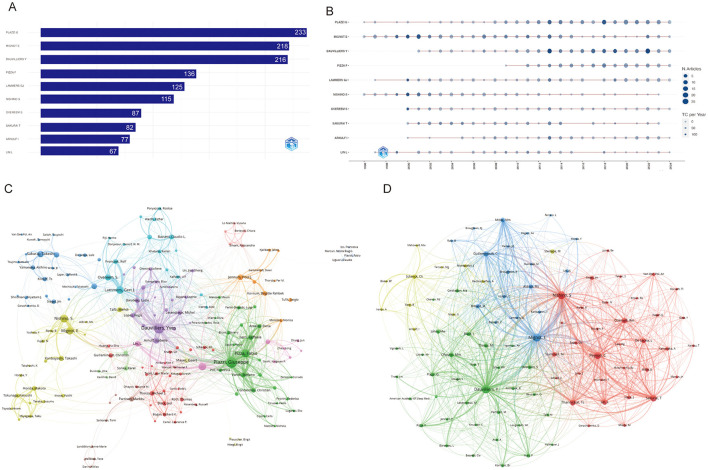
Visualization and analysis of author networks and co-cited authors in narcolepsy. **(A)** Top 10 authors by publication output. **(B)** Annual publication trends of top 10 authors. **(C)** Co-author network and **(D)** Co-cited author clustering analysis in narcolepsy research. Each circle represents an author, with lines indicating collaborative ties. The different colors of the circles denote collaborative clusters among authors. Node sizes in this network reflect the frequency of co-citations.

Altogether 19,390 authors were associated with publications related to narcolepsy (Supplementary Table 1). From 1996 to 2024, a total of 1,930 distinct authors have made contributions. Notably, the productivity of the leading authors varies considerably, with MIGNOT E and DAUVILLIERS Y ranking highest in terms of both the total number of articles published and overall citation counts. MIGNOT E, in particular, demonstrates an exceptional h-index of 78, g-index of 146, and total citations exceeding 22,000, highlighting his substantial impact on the field (Table 1). Similarly, DAUVILLIERS Y and PLAZZI G also exhibit high productivity and influence, reflected by their h-index values of 60 and 56, respectively, as well as significant citation metrics (Figure 4A; Table 1). Figure 4B illustrates the temporal distribution of publications among the top authors, showcasing consistent contributions across several decades. Authors such as MIGNOT E and PLAZZI G have maintained a steady publication rate since the mid-1990s, with notable peaks in productivity and citation rates over the years. This indicates sustained engagement and influence in their respective areas of research. Co-authorship and co-citation network analyses, depicted in Figures 4C, D, provide further insights into the collaboration and intellectual structure of the field. The co-authorship network (Figure 4C) shows clusters of collaboration, with key figures like DAUVILLIERS Y and PLAZZI G acting as central nodes, suggesting their roles as major connectors between different research groups. The presence of several distinct clusters suggests a diverse and interconnected research community, with various subgroups contributing to specific subfields. On the other hand, the co-citation network (Figure 4D) demonstrates how certain authors are frequently cited together, indicating their collective influence on foundational concepts within the discipline. The densely connected nodes within this network, particularly around MIGNOT E, reflect the foundational impact of these authors on the development of the field's theoretical and empirical knowledge base.

**Table 1:** Top 10 authors on narcolepsy research (1996–2024).

**Rank**	**Author**	**h_index**	**g_index**	**m_index**	**TC**	**NP**	**PY_start**	**Articles**	**Articles fractionalized**
1	Mignot E	78	146	2.69	22,381	218	1996	218	38.55
2	Dauvilliers Y	60	98	2.5	11,675	216	2001	216	33.98
3	Plazzi G	56	90	1.931	9,913	233	1996	233	31.90
4	Sakurai T	52	82	2.08	10,193	82	2000	82	20.20
5	Nishino S	48	112	1.655	12,714	115	1996	115	26.52
6	Lammers GJ	46	100	1.643	10,240	125	1997	125	16.62
7	Arnulf I	42	71	1.68	5,188	77	2000	77	13.39
8	Lin L	41	67	1.464	7,368	67	1997	67	7.31
9	Yanagisawa M	41	62	1.577	12,902	62	1999	62	8.50
10	Overeem S	40	87	1.6	8,656	87	2000	87	12.03

### 3.5 Journals and co-journals

**Figure 5:**
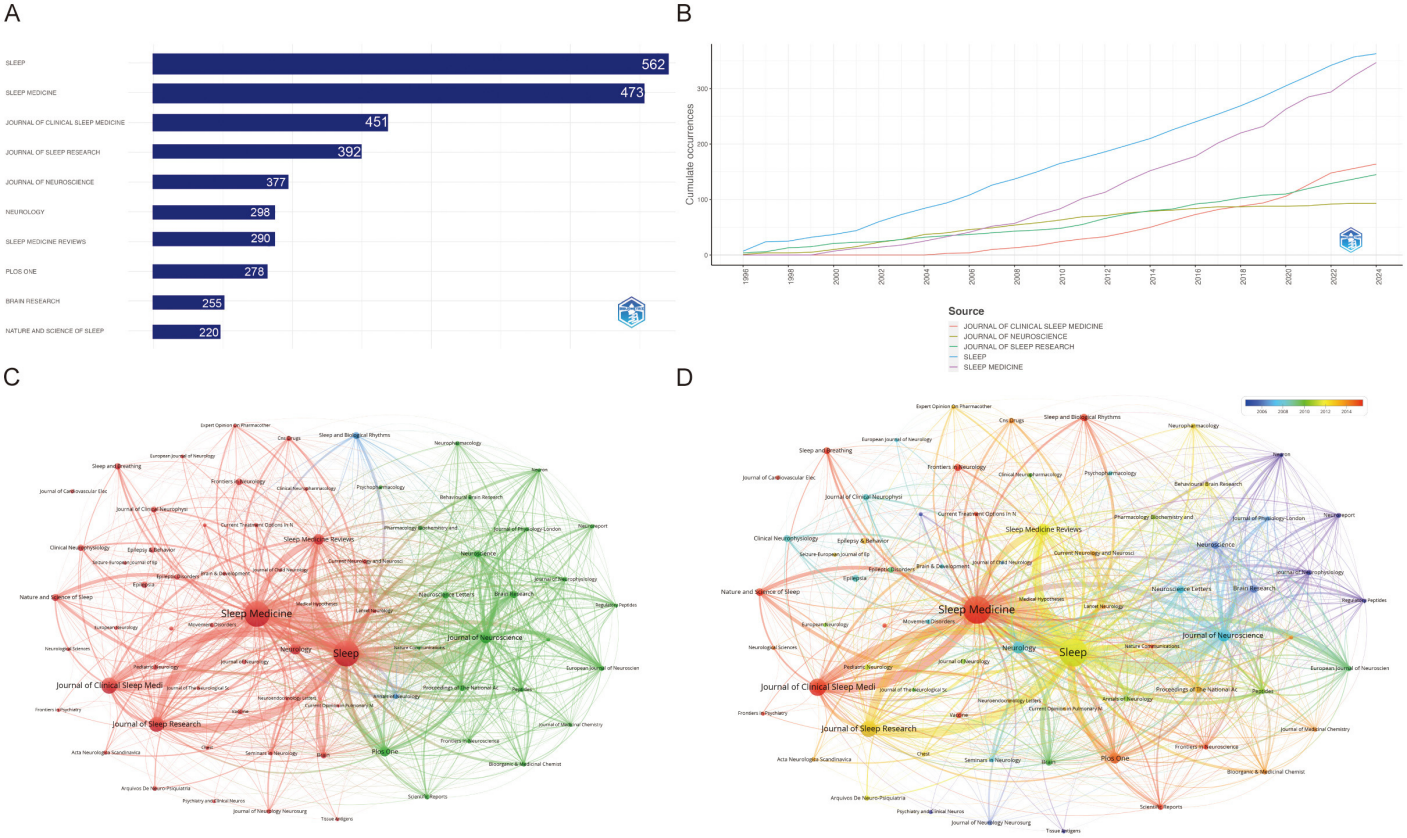
Visualization of journal association networks in narcolepsy research. **(A)** Top 10 journals by productivity. **(B)** Annual analysis of top 10 journals' production. **(C)** Co-journal clustering analysis and **(D)** Chronological timeline of research publications. Each circle represents a journal. Circle size corresponds to the strength of connections, citation counts, and other metrics. Circle colors indicate the cluster affiliation.

As can be seen from Figure 5A, it is clear that the journal ***Sleep*** has the most papers (363, 6.96%), followed by ***Sleep Medicine*** (347, 2.63%). These two journals clearly dominate the field, indicating their role as central platforms for disseminating sleep research. The determination of the top 10 most influential journals is contingent upon a multitude of parameters. The publication with the most citations is *Sleep* (20,455), indicating that it has a significant impact in this category, followed by the ***Journal of Neuroscience*** (12,958) and ***Sleep medicine*** (10,022). Sleep Medicine Reviews, with an impact factor (IF) of 12.3, holds the highest rank in its respective field. This indicates that the journal has a significant influence in the academic community, reflecting the high frequency of citations of articles published in the journal over the last 2 years (Table 2). Figure 5B illustrates the cumulative occurrence of articles published in these key journals over time. The cumulative trajectory underscores a consistent growth in the literature across leading journals, notably ***Sleep***, ***Journal of Clinical Sleep Medicine***, and ***Sleep Medicine***. From the late 1990s to 2023, the continuous rise in publications reflects the expanding interest and development in sleep-related research, with an accelerating trend observed particularly in the last decade. Figures 5C, D represent the co-citation networks of these journals, offering insights into inter-journal relationships. The network visualization (Figure 5C) demonstrates that ***Sleep*** and ***Sleep Medicine*** are pivotal nodes, heavily interconnected with other prominent journals, such as ***Journal of Clinical*** ***Sleep Medicine*** and ***Journal of Sleep Research***. The red and green clusters indicate thematic groupings, suggesting that journals within each cluster often co-publish related topics, forming distinct research subfields. Further, Figure 5D provides a temporal overlay of co-citation relationships, illustrating the evolution of these networks over time. The visualization reveals a notable expansion of interconnectivity starting around 2006, indicating a growing interdisciplinary collaboration. The color gradient, moving from yellow to red, signifies a more recent concentration of co-citations in journals like ***Sleep Medicine*** and ***Journal of Sleep Research***, reinforcing the trend toward increased collaboration in recent years. This dynamic network structure highlights the emergence of integrated research themes, pointing toward an evolving and more interconnected research landscape in the field of sleep science.

**Table 2:** Top 15 journals by influence rank according to bibliometrix analysis (1996–2024).

**Rank**	**Source**	**h_index**	**g_index**	**m_index**	**TC**	**NP**	**PY_start**	**Articles**	**JCR**	**IF**
1	Sleep	76	125	2.621	20,455	363	1996	363	Q1	5.3
2	Journal of Neuroscience	62	93	2.138	12,958	93	1996	93	Q1	4.4
3	Sleep Medicine	55	81	2.2	10,022	347	2000	347	Q1	3.8
4	Sleep Medicine Reviews	49	81	1.885	6,634	83	1999	83	Q1	11.2
5	Neurology	45	80	1.552	6,526	84	1996	84	Q1	7.7
6	Journal of Sleep Research	38	64	1.31	4,957	145	1996	145	Q2	3.4
7	Journal of Clinical Sleep Medicine	36	57	1.8	4,041	164	2005	164	Q2	3.5
8	Brain	30	34	1.111	5,225	34	1998	34	Q1	10.6
9	Neuroscience	29	43	1.074	4,403	43	1998	43	Q3	2.9
10	PLoS ONE	28	51	1.647	2,829	78	2008	78	Q2	2.9
11	Proceedings of the National Academy of Sciences of the United States of America	26	34	1.04	3,892	34	2000	34	Q1	9.4
12	Brain Research	25	44	0.862	1,941	46	1996	46	Q3	2.7
13	Epilepsia	23	35	0.793	1,947	35	1996	35	Q1	6.6
14	Neuroscience Letters	23	38	0.793	1,471	43	1996	43	Q3	2.5
15	Clinical Neurophysiology	21	35	0.808	1,285	35	1999	35	Q1	3.7

### 3.6 References and articles

The top 10 most frequently cited documents are presented in Table 3. “Narcolepsy in orexin knockout mice: molecular genetics of sleep regulation (2,372 citations)” (17) is the most cited article in the field of narcolepsy. This study reveals that orexin-deficient mice display narcolepsy-like symptoms, paralleling human patients and canarc-1 dogs. Modafinil, which activates these neurons, suggests orexin's role in narcolepsy, indicating potential therapeutic targets. This research provides a valuable animal model for understanding the molecular mechanisms underlying narcolepsy and offers insights into the role of orexin in sleep regulation. It also elucidates the potential therapeutic targets for narcolepsy, paving the way for the development of more effective treatments. The publication “International Classification of Sleep Disorders—Third Edition: Highlights and Modifications” has garnered 1,988 citations and has established diagnostic criteria and classification for narcolepsy, providing a foundation for clinical diagnosis by physicians (18). “The sleep disorder canine narcolepsy is caused by a mutation in the hypocretin receptor 2 gene,” which has been cited 1,977 times, ranks as the third most influential (19). In this research, scientists employed positional cloning to identify an autosomal recessive mutation responsible for narcolepsy in a canine model. Their discovery that the etiology of the disorder is a disruption in the hypocretin receptor 2 gene (Hcrtr2) underscores the significance of hypocretins in sleep regulation and paves the way for novel therapeutic strategies for narcolepsy in humans.

**Table 3:** Top 10 most cited publications based on bibliometrix analysis (1996–2024).

**Rank**	**Author**	**Title**	**Journal**	**Years**	**DOI**	**Total citations**	**TC per year**	**Normalized TC**
1	Chemelli RM	Narcolepsy in orexin knockout mice: molecular genetics of sleep regulation	Cell	1999	10.1016/S0092-8674(00)81973-X	2,372	91.23	20.41
2	Sateia MJ	International classification of sleep disorders-third edition: highlights and modifications	Chest	2014	10.1378/chest.14-0970	1,988	180.73	43.76
3	Lin L	The sleep disorder canine narcolepsy is caused by a mutation in the hypocretin (orexin) receptor 2 gene	Cell	1999	10.1016/S0092-8674(00)81965-0	1,977	76.04	17.01
4	Saper CB	Hypothalamic regulation of sleep and circadian rhythms	Nature	2005	10.1038/nature04284	1,804	90.20	20.80
5	Peyron C	A mutation in a case of early onset narcolepsy and a generalized absence of hypocretin peptides in human narcoleptic brains	Nat Med	2000	10.1038/79690	1,583	63.32	11.90
6	Thannickal TC	Reduced number of hypocretin neurons in human narcolepsy	Neuron	2000	10.1016/S0896-6273(00)00058-1	1,549	61.96	11.64
7	Nishino S	Hypocretin (orexin) deficiency in human narcolepsy	Lancet	2000	10.1016/S0140-6736(99)05582-8	1,329	53.16	9.99
8	Marcus JN	Differential expression of orexin receptors 1 and 2 in the rat brain	J Comp Neurol	2001	10.1002/cne.1190	1,267	52.79	10.26
9	Kushida CA	Practice parameters for the indications for polysomnography and related procedures: an update for 2005	Sleep	2005	10.1093/sleep/28.4.499	1,146	57.30	13.21
10	Hara J	Genetic ablation of orexin neurons in mice results in narcolepsy, hypophagia, and obesity	Neuron	2001	10.1016/S0896-6273(01)00293-8	1,098	45.75	8.89

**Figure 6:**
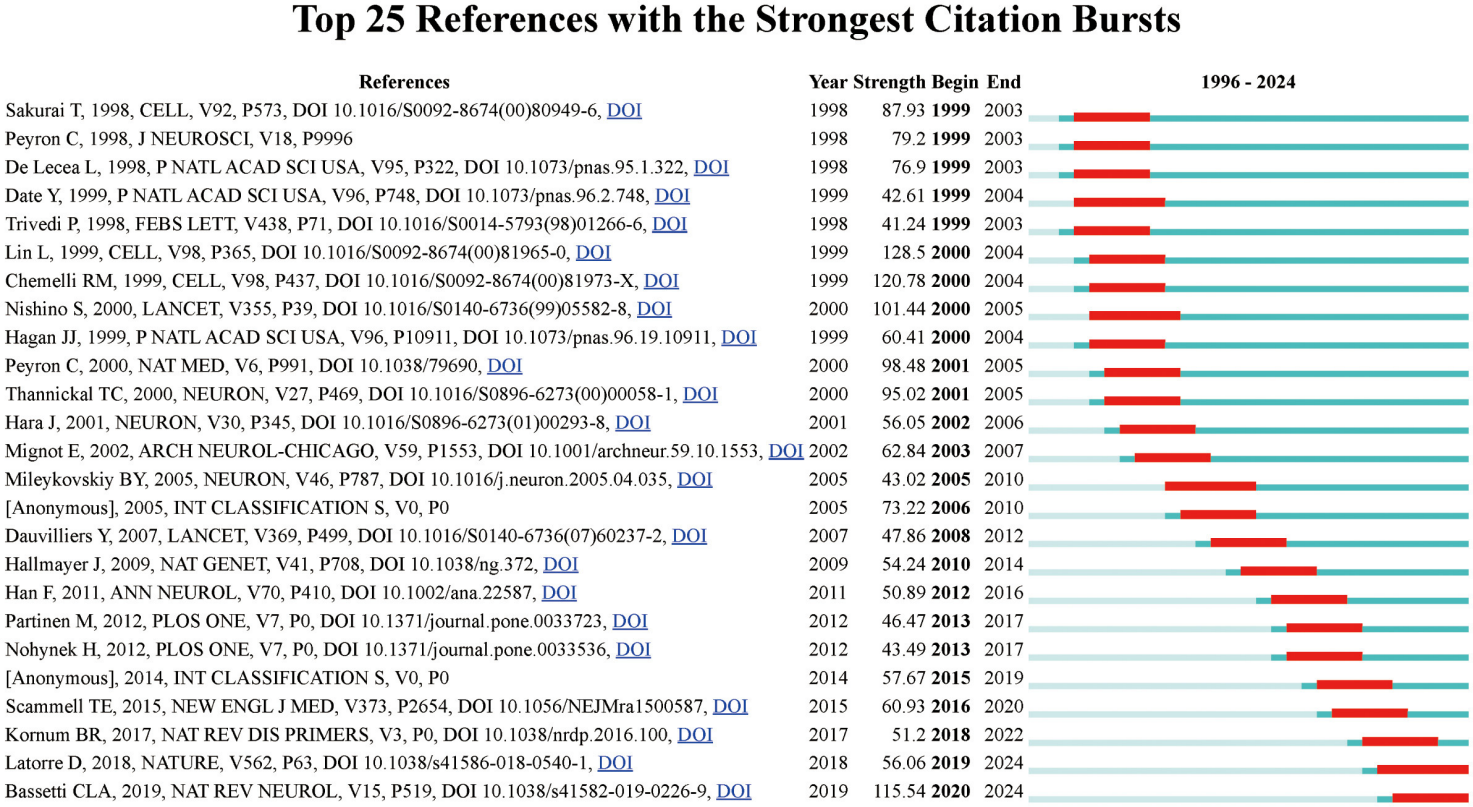
Top 25 references with the strongest citation bursts in narcolepsy research. The blue lines indicate the time intervals, while red lines mark the periods of burst activity for each reference, showing the initial and final years of the citation burst duration.

Figure 6 shows the main 25 references with the most grounded burst force and ranks them in chronological order. providing insights into the research articles that have significantly influenced the field at various points in time. Notably, Sakurai's 1998 article (20), which identifies the link between hypocretin and sleep regulation, exhibited the highest citation burst strength from 1999 to 2003, emphasizing its critical contribution to understanding sleep mechanisms. Other key papers, such as Peyron et al. (21) and de Lecea (22), showed intense citation activity during similar periods, further reinforcing the foundational nature of discoveries related to the orexin neuropeptide system. Additionally, the timeline reveals bursts of citations in the late 2000s and early 2010s, corresponding to discoveries in narcolepsy genetics, vaccination research, and the role of potassium in sleep regulation. Such bursts highlight periods of heightened academic focus and rapid progress in specific domains of sleep research, providing a temporal understanding of the evolving research frontiers in the field.

### 3.7 Keyword analysis

**Figure 7:**
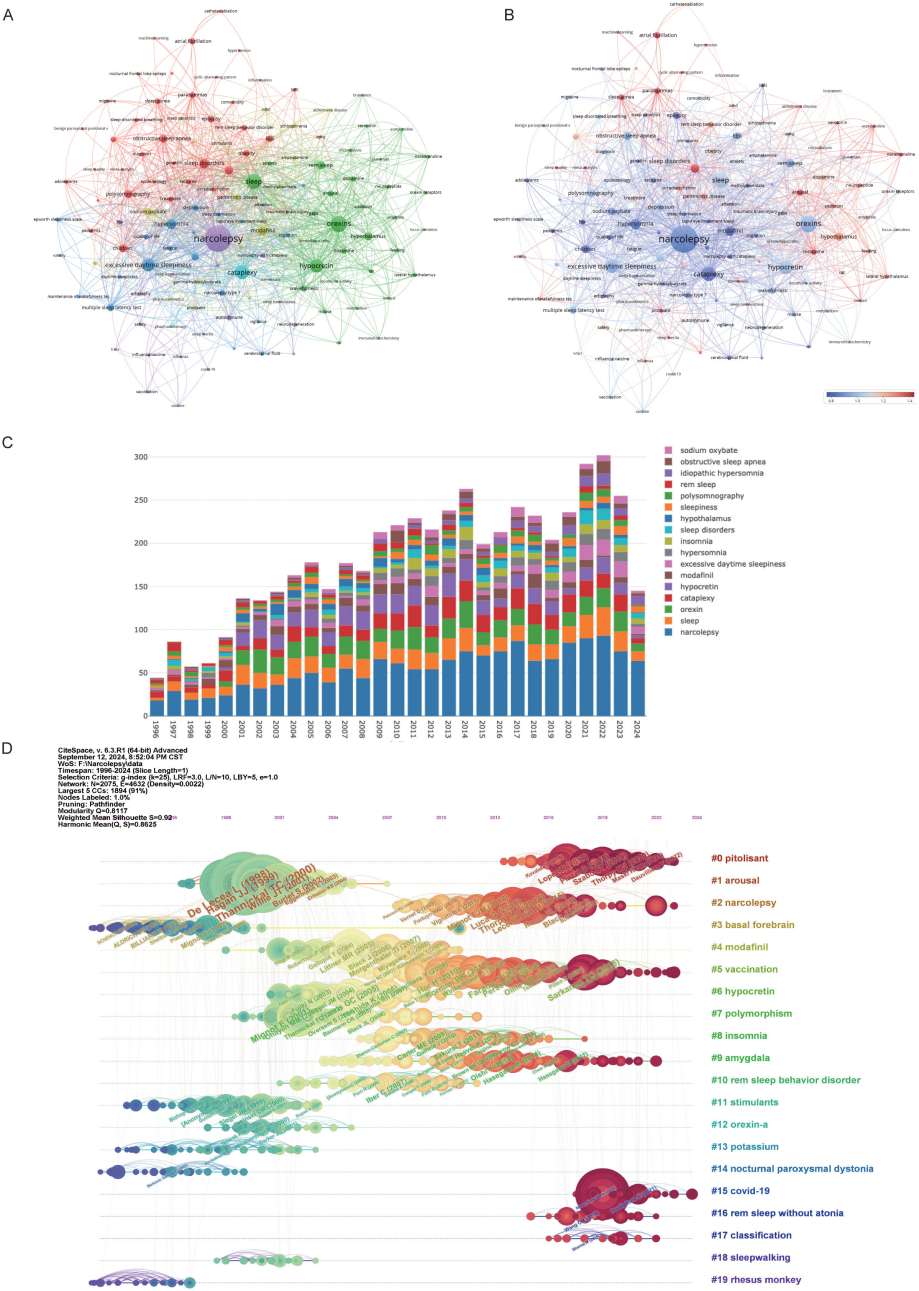
Visualization of keywords in narcolepsy research. **(A)** Co-occurrence Clustering of Keywords and **(B)** Average Normal Citations in Author-Keyword Analysis. Each circle and its associated label represent a keyword unit, with different colors indicating distinct clusters. **(C)** Annual trends in keyword mentions. **(D)** Timeline of keyword evolution in narcolepsy.

Figure 7A reveals the intricate relationships between key research terms in narcolepsy, as visualized through a keyword co-occurrence network. In this network, individual keywords from published literature are represented as nodes, with their size corresponding to their frequency of occurrence. The edges between nodes denote co-occurrences within the same publications, reflecting thematic connections between different research topics. Several distinct clusters were identified, with each cluster highlighting specific areas of research. The central cluster focuses on core terms such as “narcolepsy”, “cataplexy” and “hypocretin”, indicating a strong emphasis on the clinical features and mechanisms of narcolepsy. Other significant clusters include neurobiological themes (e.g., “orexins,” “dopamine,” and “hypothalamus”), diagnostic tools and related sleep disorders (e.g., “polysomnography” and “obstructive sleep apnea(OSA)”, and treatment strategies, particularly focusing on “modafinil” and pharmacotherapy. Figure 7B suggests a shift in recent research focus. Older research focused on foundational concepts such as narcolepsy's clinical symptoms and underlying neurochemical mechanisms, as represented by the blue nodes and edges. More recent research trends, highlighted in red, include emerging topics like machine learning, COVID-19, and autoimmune responses, indicating a growing interest in interdisciplinary approaches that incorporate computational techniques and public health challenges. This temporal analysis demonstrates how research priorities have evolved over time, from foundational studies to a more diversified focus that includes newer societal and technological impacts. The prominence of terms such as “hypocretin” and “orexins” throughout these clusters underscores their central role in understanding the pathology and treatment of narcolepsy. The stacked bar chart confirmed the above results (Figure 7C). Figure 7D illustrates the evolution of citations alongside changes in keywords. The network, visualized using CiteSpace software, depicted distinct research clusters, with nodes representing publications and edges indicating co-citation links. A color gradient from blue to red was used to depict the temporal evolution of research, where blue indicated earlier studies and red reflected more recent ones. Key clusters emerged, such as the largest cluster on “pitolisant” which signifies recent advancements in treatments for narcolepsy. Pitolisant is a first-in-class medication that acts as a selective histamine H3 receptor antagonist/inverse agonist, enhancing histaminergic transmission in the brain, which leads to increased wakefulness. It has been approved for the treatment of EDS in adults with narcolepsy, both with and without cataplexy (23). Other clusters include foundational studies on narcolepsy's pathology, treatment, and diagnosis, as well as topics like arousal mechanisms and modafinil use. The modularity score (*Q* = 0.8117) and silhouette score (*S* = 0.8625) indicated a well-structured and consistent clustering of topics. Furthermore, against the backdrop of the COVID-19 pandemic, the association between narcolepsy and “#15 COVID-19” has also garnered significant attention, as has “#5 vaccination”.

## 4 Discussion

Narcolepsy, a relatively rare neurological condition, is characterized by persistent EDS and unpredictable sleep episodes, severely compromising the affected individual's daily functioning and overall quality of life (24). The diagnostic journey for narcolepsy is often lengthy and challenging due to the heterogeneity of symptoms and potential overlap with other disorders (24, 25). This ambiguity can further prolong diagnosis. Additionally, the financial burden of managing narcolepsy is significant, imposing substantial economic strain on patients (26). The prevalence of comorbid conditions is also increased in this population, compounding the complexity of treatment and the daily challenges faced by individuals with the disorder (27).

Visualizing the frequency and evolution of keywords in the literature included in the study, we found that the main keywords cover four aspects: molecular pathogenesis based on key neurotransmitters, comorbidities, symptomatology, and treatment. Undiagnosed EDS and narcolepsy are common, with many cases overlooked due to symptom overlap with conditions such as attention deficit hyperactivity disorder and epilepsy, resulting in frequent misdiagnosis (28, 29). Consequently, insufficient attention is paid to symptomatology. The main keywords include “narcolepsy,” “cataplexy,” “hypersomnia,” “wakefulness,” “vigilance,” “excessive daytime sleepiness” and “insomnia.” etc. A confirmed diagnosis of narcolepsy after a period of being undiagnosed has been associated with reduced medical visits, from 28.2 to 22.5 per year, and an increase in narcolepsy -related medication use from 54.0% to 66.9% by year 3 postdiagnosis (30). This highlights the importance of timely diagnosis, as it improves patient management and enhances quality of life.

The molecular pathogenesis primarily focuses on the molecular mechanisms associated with hypocretin. Hypocretin, also known as orexin, is a neuropeptide produced by neurons in the hypothalamus and plays a crucial role in regulating sleep and wakefulness (20). In patients with narcolepsy, the loss of hypocretin neurons leads to reduced levels of hypocretin, potentially due to immune reactions, genetic factors, or environmental influences. Hypocretin regulates arousal and energy levels by interacting with various neural pathways in the brain, including those involving dopamine, norepinephrine, histamine, and acetylcholine (22). Thus, the keywords encompass significant terms within the field, including hypothalamus, autoimmune, dopamine receptors, neuropeptide, glutamate, dopamine, and acetylcholine. Hypocretin significantly impacts dopamine neurons, particularly in the ventral tegmental area, crucial for motivated behavior and reward processing. Activation of orexin 1 receptors enhances dopamine firing, while kappa opioid receptor activation has the opposite effect, indicating nuanced modulation of reward-seeking behavior (31, 32). However, In NT1, elevated dopamine levels coincide with reduced orexin-A levels, indicating a detrimental effect of narcolepsy on orexins (33). This suggests a complex interplay between these neurotransmitters, although the exact nature of their relationship—whether those neurotransmitters influence orexins or vice versa—remains to be clarified. Immune mechanisms play a crucial role in the pathogenesis of NT1, as evidenced by a strong association with the MHC class II allele HLA-DQB1^*^06:02, suggesting a genetic predisposition to immune system involvement (34). Additionally, increased T cell reactivity against hypocretin, a neuropeptide essential for regulating wakefulness, indicates that the immune system may mistakenly target and destroy hypocretin-producing neurons (35). The higher incidence of NT1 following Pandemrix influenza vaccination further supports the involvement of immunopathogenesis, suggesting that immune responses triggered by the vaccine may contribute to disease onset (36). Further understanding of this interaction, particularly through *in vivo* and *in vitro* experiments, could provide valuable insights into the mechanisms underlying narcolepsy.

Furthermore, increasing attention to the quality of life of narcolepsy patients has led to the emergence of keywords related to comorbidities, such as “obesity,” “OSA,” “comorbidity,” “migraine,” “hypertension,” and “atrial fibrillation (AF),”among others. Narcolepsy, particularly in pediatric patients, is significantly associated with an elevated risk of developing OSA, with an odds ratio of 4.87 (*P* < 0.001) (37). In adults, the presence of OSA can complicate the diagnosis of narcolepsy, as demonstrated in a case where severe OSA obscured the symptoms of NT1 (38). Furthermore, OSA is known to contribute to EDS, a hallmark symptom of NT1. Recent advancements in machine learning have facilitated the development of models capable of predicting a high probability of comorbid NT1 in patients with OSA, thereby indicating a potential link between these two sleep disorders (39). This interplay suggests that clinicians should be vigilant in assessing for both conditions, as the presence of one may influence the diagnosis and management of the other. Narcolepsy has also been linked with AF, as evidenced by a study in Slovakia where 5% of narcoleptic patients were found to have AF, although this association remains not fully established (40). Furthermore, individuals with narcolepsy are at increased risk of cardiovascular events, including AF, with a reported hazard ratio of 1.48 when compared to non-narcoleptic individuals (41). This suggests that while the direct relationship between narcolepsy and AF requires further investigation, there is a notable correlation that warrants attention in clinical settings. Narcolepsy also is associated with higher odds of anxiety disorders (OR 1.381, 95% CI 1.161–1.642, *p* < 0.001) (42) and significantly linked to depression (OR 1.055, 95% CI 1.015–1.097) (43). Taken together, these findings emphasize the complexity of narcolepsy as a disorder that extends beyond the core symptoms of EDS and cataplexy. The interplay between narcolepsy and its comorbidities—ranging from OSA and AF to obesity, anxiety, depression, and migraine—underscores the necessity for a holistic approach to care. Early identification and treatment of comorbid conditions are paramount to improving outcomes for patients with narcolepsy.

To date, pharmacotherapy for narcolepsy has advanced significantly, with new treatments targeting EDS and cataplexy now either approved or in clinical trials. Modafinil, a wake-promoting agent, has been shown to be effective in treating EDS associated with narcolepsy, particularly in females, without negatively impacting nighttime sleep quality (44). However, the comparative effectiveness of modafinil to traditional stimulants like amphetamines remains to be fully elucidated (45). Sodium Oxybate (ON-SXB) has been shown to significantly improve EDS and other symptoms in patients with narcolepsy, enhancing sleep latency and reducing weight in clinical trials (46). However, its use requires caution due to potential severe side effects, including respiratory failure and an increased risk of hypertension (47). In cases where modafinil is ineffective, such as idiopathic hypersomnia, ON-SXB has emerged as a potential alternative, indicating its role when first-line treatments fail (48). Pitolisant, the only FDA-approved histamine H3 receptor antagonist for narcolepsy, has demonstrated promise in alleviating symptoms such as EDS and REM sleep disturbances (49). Notably, pitolisant has been effective in supporting drug holidays for patients with tolerance to modafinil, leading to significant reductions in modafinil dosage and improvements in EDS and health-related quality of life (50). Furthermore, it has proven beneficial for patients who experienced inadequate responses or adverse effects from ON-SXB, allowing for a reduced dosage of ON-SXB and minimizing associated side effects. In patients with OSA, both modafinil and pitolisant have demonstrated efficacy in reducing EDS, with pitolisant showing a notable decrease in the Epworth Sleepiness Scale score, however, direct comparisons between the two treatments are lacking (50). Thus, the evolving pharmacotherapy of narcolepsy reflects a shift toward more targeted and personalized treatments, encompassing both symptomatic relief and, potentially, disease-modifying approaches.

Our study offers several key strengths. As one of the first to systematically examine the narcolepsy research landscape using bibliometric methods, it provides a quantitative assessment of the field's evolution. The study's broad literature coverage delivers a comprehensive overview, while the use of tools like VOSviewer and CiteSpace enables objective mapping of research trends, key contributors, and knowledge gaps. These methods enhance the reproducibility and robustness of the analysis, offering valuable insights to guide future research directions. However, several limitations must be acknowledged. The focus on English-language publications may underrepresent research from non-English-speaking regions, limiting the global scope. Inconsistencies in author names and institutional affiliations may also affect data accuracy. Lastly, the inherent time lag between data collection and publication could result in the omission of the most recent studies.

In summary, our study provides valuable insights into the progression of narcolepsy research, highlighting the need for ongoing focus on symptomatology, comorbidities, and the development of novel therapeutic strategies to improve the quality of life for individuals with narcolepsy. The interactions between key neurotransmitters are complex, and further research is needed to elucidate the precise mechanisms underlying these interactions and their implications for sleep regulation and behavior.

## 5 Conclusion

Our study highlights the significant advances in narcolepsy research over the past two decades and reveals a dynamic, evolving landscape. Future directions are likely to emphasize the role of the immune system, the development of innovative non-stimulant treatments, and a more comprehensive understanding of the mental health and quality-of-life challenges faced by individuals living with narcolepsy.

## Data Availability

The raw data supporting the conclusions of this article will be made available by the authors, without undue reservation.

## References

[1] Bassetti C Aldrich MS. Narcolepsy. Neurol Clin. (1996) 14:545–71. 10.1016/S0733-8619(05)70273-5 8871976

[2] Scammell TE. Narcolepsy. N Engl J Med. (2015) 373:2654–62. 10.1056/NEJMra1500587 26716917

[3] Barateau L Pizza F Plazzi G Dauvilliers Y. Narcolepsy. J Sleep Res. (2022) 31:e13631. 10.1111/jsr.13631 35624073

[4] Thorpy MJ Krieger AC. Delayed diagnosis of narcolepsy: characterization and impact. Sleep Med. (2014) 15:502–7. 10.1016/j.sleep.2014.01.015 24780133

[5] Mahoney CE Cogswell A Koralnik IJ Scammell TE. The neurobiological basis of narcolepsy. Nat Rev Neurosci. (2019) 20:83–93. 10.1038/s41583-018-0097-x 30546103 PMC6492289

[6] Zhang Y Ren R Yang L Zhang H Shi Y Sanford LD . Polysomnographic nighttime features of narcolepsy: A systematic review and meta-analysis. Sleep Med Rev. (2021) 58:101488. 10.1016/j.smrv.2021.101488 33934047

[7] Mcburney MK Novak PL. What is bibliometrics and why should you care?. In: Proceedings. IEEE International Professional Communication Conference: IEEE (2002). p. 108–114.

[8] Baryah N Krishan K Kanchan T. Bibliometrics and scientometrics: evaluating the research. J Indian Acad Foren Med. (2020) 42:150–2. 10.5958/0974-0848.2020.00041.X

[9] Marginson S. Global science and national comparisons: beyond bibliometrics and scientometrics. Compar Educ. (2021) 58:125–46. 10.1080/03050068.2021.1981725

[10] Adams J. Bibliometrics: the citation game. Nature. (2014) 510:470–1. 10.1038/510470a

[11] Alryalat SAS Malkawi LW Momani SM. Comparing bibliometric analysis using pubmed, scopus, and web of science databases. J Vis Exp. (2019) 152:e58494 10.3791/58494 31710021

[12] Chen C. CiteSpace II: Detecting and visualizing emerging trends and transient patterns in scientific literature. J Am Soc Inform Sci Technol. (2006) 57:359–77. 10.1002/asi.20317

[13] Van Eck NJ Waltman L. Software survey: VOSviewer, a computer program for bibliometric mapping. Scientometrics. (2010) 84:523–38. 10.1007/s11192-009-0146-3 20585380 PMC2883932

[14] Aria M Cuccurullo C. bibliometrix: An R-tool for comprehensive science mapping analysis. J Informetr. (2018) 11:959–75. 10.1016/j.joi.2017.08.007

[15] Egghe L. Theory and practise of the g-index. Scientometrics. (2006) 69:131–52. 10.1007/s11192-006-0144-7

[16] Hirsch JE. An index to quantify an individual's scientific research output. Proc Nat Acad Sci. (2005) 102:16569–72. 10.1073/pnas.0507655102 16275915 PMC1283832

[17] Chemelli RM Willie JT Sinton CM Elmquist JK Scammell T Lee C . Narcolepsy in orexin knockout mice: molecular genetics of sleep regulation. Cell. (1999) 98:437–51. 10.1016/S0092-8674(00)81973-X 10481909

[18] Sateia MJ. International classification of sleep disorders-third edition: highlights and modifications. Chest. (2014) 146:1387–94. 10.1378/chest.14-0970 25367475

[19] Lin L Faraco J Li R Kadotani H Rogers W Lin X . The sleep disorder canine narcolepsy is caused by a mutation in the hypocretin (orexin) receptor 2 gene. Cell. (1999) 98:365–76. 10.1016/S0092-8674(00)81965-0 10458611

[20] Sakurai T Amemiya A Ishii M Matsuzaki I Chemelli RM Tanaka H . Orexins and orexin receptors: a family of hypothalamic neuropeptides and G protein-coupled receptors that regulate feeding behavior. Cell. (1998) 92:573–85. 10.1016/S0092-8674(00)80949-6 9491897

[21] Peyron C Tighe DK Van Den Pol AN De Lecea L Heller HC Sutcliffe JG . Neurons containing hypocretin (orexin) project to multiple neuronal systems. J Neurosci. (1998) 18:9996–10015. 10.1523/JNEUROSCI.18-23-09996.1998 9822755 PMC6793310

[22] De Lecea L Kilduff TS Peyron C Gao X Foye PE Danielson FS . The hypocretins: hypothalamus-specific peptides with neuroexcitatory activity. Proc Natl Acad Sci U S A. (1998) 95:322–7. 10.1073/pnas.95.1.322 9419374 PMC18213

[23] Lamb YN. Pitolisant: a review in narcolepsy with or without cataplexy. CNS Drugs. (2020) 34:207–18. 10.1007/s40263-020-00703-x 31997137

[24] Bassetti CLA Adamantidis A Burdakov D Han F Gay S Kallweit U . Narcolepsy—clinical spectrum, aetiopathophysiology, diagnosis and treatment. Nat Rev Neurol. (2019) 15:519–39. 10.1038/s41582-019-0226-9 31324898

[25] Dauvilliers Y Arnulf I Mignot E. Narcolepsy with cataplexy. Lancet. (2007) 369:499–511. 10.1016/S0140-6736(07)60237-2 17292770

[26] Kamada Y Imanishi A Chiu SW Yamaguchi T. Burden of narcolepsy in Japan: a health claims database study evaluating direct medical costs and comorbidities. Sleep Med. (2024) 114:119–27. 10.1016/j.sleep.2023.12.020 38181583

[27] Morse AM Kim SY Harris S Gow M. Narcolepsy: beyond the classic pentad. CNS Drugs. (2025) 39:9–22. 10.1007/s40263-024-01141-9 40111737 PMC11950085

[28] Rains JC. Sleep and migraine: assessment and treatment of comorbid sleep disorders. Headache. (2018) 58:1074–91. 10.1111/head.13357 30095163

[29] Naya N Tsuji T Nishigaki N Sakai C Chen Y Jung S . The burden of undiagnosed adults with attention-deficit/hyperactivity disorder symptoms in Japan: a cross-sectional study. Cureus. (2021) 13:e19615. 10.7759/cureus.19615 34956750 PMC8674614

[30] Villa KF Reaven NL Funk SE Mcgaughey K Black J. Changes in medical services and drug utilization and associated costs after narcolepsy diagnosis in the United States. Am Health Drug Benefits. (2018) 11:137–45. 29910845 PMC5973250

[31] Mohammadkhani A Mitchell C James MH Borgland SL Dayas CV. Contribution of hypothalamic orexin (hypocretin) circuits to pathologies of motivation. Br J Pharmacol. (2024) 181:4430–49. 10.1111/bph.17325 39317446 PMC11458361

[32] Mohammadkhani A Qiao M Borgland SL. Distinct neuromodulatory effects of endogenous orexin and dynorphin corelease on projection-defined ventral tegmental dopamine neurons. J Neurosci. (2024) 44:e0682242024. 10.1101/2024.08.01.606179 39187377 PMC11426376

[33] Xu W Zhang X Zhang B Lu S Huang W Xu J . Clinical features and mechanisms of neck myoclonus in narcolepsy. Sleep Med. (2024) 123:22–8. 10.1016/j.sleep.2024.08.024 39226673

[34] Liblau RS Latorre D Kornum BR Dauvilliers Y Mignot EJ. The immunopathogenesis of narcolepsy type 1. Nat Rev Immunol. (2024) 24:33–48. 10.1038/s41577-023-00902-9 37400646

[35] Ollila HM Sharon E Lin L Sinnott-Armstrong N Ambati A Yogeshwar SM . Narcolepsy risk loci outline role of T cell autoimmunity and infectious triggers in narcolepsy. Nat Commun. (2023) 14:2709. 10.1038/s41467-023-36120-z 37188663 PMC10185546

[36] Pagh-Berendtsen N Pavlovskyi A Flores Téllez D Egebjerg C Kolmos MG Justinussen J . Downregulation of hypocretin/orexin after H1N1 Pandemrix vaccination of adolescent mice. Sleep. (2024) 47:zsae014. 10.1093/sleep/zsae014 38227834

[37] Geng C Chen C. Estimating the prevalence and clinical causality of obstructive sleep apnea in paediatric narcolepsy patients. Sleep Breath. (2024) 28:2147–53. 10.1007/s11325-024-03100-6 38985234

[38] Martinet V Sinkunaite L Bruyneel M. An Unusual cause of increasing excessive daytime sleepiness in a CPAP-treated obstructive sleep apnea patient. Sleep Sci. (2024) 17:e208–11. 10.1055/s-0043-1777832 38846592 PMC11152635

[39] Pan Y Zhao D Zhang X Yuan N Yang L Jia Y . Machine learning-based model for prediction of narcolepsy type 1 in patients with obstructive sleep apnea with excessive daytime sleepiness. Nat Sci Sleep. (2024) 16:639–52. 10.2147/NSS.S456903 38836216 PMC11149636

[40] Feketeova E Tormasiova M Klobučníková K Durdik P Jarcuskova D Benca M . Narcolepsy in Slovakia—epidemiology, clinical and polysomnographic features, comorbid diagnoses: a case-control study. Sleep Med. (2020) 67:15–22. 10.1016/j.sleep.2019.10.012 31884306

[41] Ben-Joseph RH Saad R Black J Dabrowski EC Taylor B Gallucci S . Cardiovascular Burden of Narcolepsy Disease (CV-BOND): a real-world evidence study. Sleep. (2023) 46:zsad161. 10.1093/sleep/zsad161 37305967 PMC10566243

[42] Geng C Chen C. Causal relationship between narcolepsy and anxiety: a two-sample Mendelian randomization study. J Psychosom Res. (2024) 182:111802. 10.1016/j.jpsychores.2024.111802 38762991

[43] Jin Y. Causal relationship between narcolepsy and depression: a two-sample Mendelian randomization study. J Psychosom Res. (2023) 175:111517. 10.1016/j.jpsychores.2023.111517 37832275

[44] US Modafinil in Narcolepsy Multicenter Study Group. Randomized trial of modafinil for the treatment of pathological somnolence in narcolepsy. Ann Neurol (1998) 43:88–97. 10.1002/ana.410430115 9450772

[45] Christensen J Vlassopoulos E Barlow CK Schittenhelm RB Li CN Sgro M . The beneficial effects of modafinil administration on repeat mild traumatic brain injury (RmTBI) pathology in adolescent male rats are not dependent upon the orexinergic system. Exp Neurol. (2024) 382:114969. 10.1016/j.expneurol.2024.114969 39332798

[46] Roth T Morse AM Bogan R Roy A Gudeman J Dauvilliers Y . Weight loss with once-nightly sodium oxybate for the treatment of narcolepsy: analysis from the phase III randomized study evaluating the efficacy and SafeTy of a ONce nightly formulation of sodium oxybate (REST-ON) Trial. Clin Ther. (2024) 46:791–8. 10.1016/j.clinthera.2024.07.010 39153911

[47] Mcintosh BW Mayeux C. Accidental calcium, magnesium, potassium and sodium oxybates (Xywave) overdose: mistiming of a single night's narcolepsy medication leading to respiratory failure requiring mechanical ventilation. BMJ Case Rep. (2024) 17:e260025. 10.1136/bcr-2024-260025 38821567

[48] Vera Ricaurte M Akhtar J Patel P Sundaram A Kharel KK Kagzi M . The role of sodium oxybate in idiopathic hypersomnia: a case report showing improvement of excessive daytime sleepiness and reduced symptoms. Cureus. (2023) 15:e45976. 10.7759/cureus.45976 37900508 PMC10600639

[49] Gao M Ooms JF Leurs R Vischer HF. Histamine H(3) receptor isoforms: insights from alternative splicing to functional complexity. Biomolecules. (2024) 14:761. 10.3390/biom14070761 39062475 PMC11274711

[50] Winter Y Lang C Kallweit U Apel D Fleischer V Ellwardt E . Pitolisant-supported bridging during drug holidays to deal with tolerance to modafinil in patients with narcolepsy. Sleep Med. (2023) 112:116–21. 10.1016/j.sleep.2023.10.005 37839272

