# Correction: Narcolepsy: a machine learning bibliometric analysis (1996–2024)

**DOI:** 10.3389/fneur.2025.1659391

**Published:** 2025-07-25

**Authors:** Minheng Zhang, Xiaodong Hu, Haiyan Wu, Haixia Fan

**Affiliations:** ^1^The First People's Hospital of Jinzhong, Jinzhong, Shanxi, China; ^2^First Hospital of Shanxi Medical University, Taiyuan, Shanxi, China

**Keywords:** narcolepsy, comorbidities, molecular mechanisms, treatment, bibliometric analysis

Author Minheng Zhang was erroneously assigned to affiliation First Hospital of Shanxi Medical University, Taiyuan, Shanxi, China. This affiliation has now been removed for author Minheng Zhang.

Authors Xiaodong Hu and Haixia Fan were erroneously assigned to affiliation The First People's Hospital of Jinzhong, Jinzhong, Shanxi, China. This affiliation has now been removed for authors Xiaodong Hu and Haixia Fan

The correct order of affiliations should be as follows:

Minheng Zhang^1^, Xiaodong Hu^2^, Haiyan Wu^2^, Haixia Fan^2^^*^

1. The First People's Hospital of Jinzhong, Jinzhong, Shanxi, China.

2. First Hospital of Shanxi Medical University, Taiyuan, Shanxi, China

The original version of this article has been updated.

