# Supplementary material for: Narcolepsy: a machine learning bibliometric analysis (1996–2024)
Source: Front Neurol. 2025 Jun 25;16:1505574. doi: 10.3389/fneur.2025.1505574 (PMC12241812; doi:10.3389/fneur.2025.1505574)
Supplement: Supplementary file 1 [file Table_1.docx]

**Supplementary Table 1** Bibliometric Summary of Narcolepsy-Related Publications (1996–2024)

| **Description** | **Results** |
| --- | --- |
| Timespan | 1996:2024 |
| Sources (Journals, Books, etc) | 1086 |
| Documents | 5215 |
| Annual Growth Rate % | 2.96 |
| Document Average Age | 11.5 |
| Average citations per doc | 44.22 |
| References | 112470 |
| DOCUMENT CONTENTS |  |
| Keywords Plus (ID) | 7445 |
| Author's Keywords (DE) | 7634 |
| AUTHORS |  |
| Authors | 17298 |
| Authors of single-authored docs | 280 |
| AUTHORS COLLABORATION |  |
| Single-authored docs | 366 |
| Co-Authors per Doc | 6.05 |
| International co-authorships % | 25.7 |
| DOCUMENT TYPES |  |
| article | 4237 |
| review | 978 |

**Supplementary Table 2.** Most Relevant Countries by Corresponding Author Contributions

| **Rank** | **Country** | **Articles** | **Articles %** | **SCP** | **MCP** | **MCP %** | **TC** | **Average Article Citations** | |
| --- | --- | --- | --- | --- | --- | --- | --- | --- | --- |
| 1 | USA | 1626 | 31.2 | 1291 | 335 | 20.6 | 104172 | 64.10 |  |
| 2 | ITALY | 421 | 8.1 | 305 | 116 | 27.6 | 13610 | 32.30 |  |
| 3 | JAPAN | 399 | 7.7 | 320 | 79 | 19.8 | 15772 | 39.50 |  |
| 4 | FRANCE | 349 | 6.7 | 219 | 130 | 37.2 | 16373 | 46.90 |  |
| 5 | CHINA | 289 | 5.5 | 232 | 57 | 19.7 | 4419 | 15.3 |  |
| 6 | UNITED KINGDOM | 222 | 4.3 | 162 | 60 | 27 | 9681 | 43.60 |  |
| 7 | GERMANY | 202 | 3.9 | 142 | 60 | 29.7 | 8592 | 42.50 |  |
| 8 | SWITZERLAND | 194 | 3.7 | 99 | 95 | 49 | 10029 | 51.70 |  |
| 9 | CANADA | 161 | 3.1 | 116 | 45 | 28 | 10751 | 66.80 |  |
| 10 | NETHERLANDS | 145 | 2.8 | 105 | 40 | 27.6 | 5742 | 39.60 |  |

Abbreviation: SCP :Single Country Publications; MCP :Multiple Country Publications; TC: Total Citation

**Supplementary Table 3**. TOP 10 Affiliations and Article Counts on Narcolepsy (1996–2024)

| **Affiliation** | **Articles** |
| --- | --- |
| STANFORD UNIVERSITY | 562 |
| INSTITUT NATIONAL DE LA SANTE ET DE LA RECHERCHE MEDICALE (INSERM) | 473 |
| UNIVERSITE DE MONTPELLIER | 451 |
| HARVARD UNIVERSITY | 392 |
| UNIVERSITY OF BOLOGNA | 377 |
| UNIVERSITY OF CALIFORNIA SYSTEM | 298 |
| ASSISTANCE PUBLIQUE HOPITAUX PARIS (APHP) | 290 |
| CHU DE MONTPELLIER | 278 |
| MAYO CLINIC | 255 |
| UNIVERSITY OF COPENHAGEN | 220 |
